# The Inotropic Effect of the Active Metabolite of Levosimendan, OR-1896, Is Mediated through Inhibition of PDE3 in Rat Ventricular Myocardium

**DOI:** 10.1371/journal.pone.0115547

**Published:** 2015-03-04

**Authors:** Øivind Ørstavik, Ornella Manfra, Kjetil Wessel Andressen, Geir Øystein Andersen, Tor Skomedal, Jan-Bjørn Osnes, Finn Olav Levy, Kurt Allen Krobert

**Affiliations:** 1 Department of Pharmacology, Faculty of Medicine, University of Oslo and Oslo University Hospital, Oslo, Norway; 2 K.G. Jebsen Cardiac Research Centre, Faculty of Medicine, University of Oslo, Oslo, Norway; 3 Center for Heart Failure Research, Faculty of Medicine, University of Oslo, Oslo, Norway; 4 Department of Cardiology, Oslo University Hospital, Oslo, Norway; University of Torino, ITALY

## Abstract

**Aims:**

We recently published that the positive inotropic response (PIR) to levosimendan can be fully accounted for by phosphodiesterase (PDE) inhibition in both failing human heart and normal rat heart. To determine if the PIR of the active metabolite OR-1896, an important mediator of the long-term clinical effects of levosimendan, also results from PDE3 inhibition, we compared the effects of OR-1896, a representative Ca^2+^ sensitizer EMD57033 (EMD), levosimendan and other PDE inhibitors.

**Methods:**

Contractile force was measured in rat ventricular strips. PDE assay was conducted on rat ventricular homogenate. cAMP was measured using RII_epac FRET-based sensors.

**Results:**

OR-1896 evoked a maximum PIR of 33±10% above basal at 1 μM. This response was amplified in the presence of the PDE4 inhibitor rolipram (89±14%) and absent in the presence of the PDE3 inhibitors cilostamide (0.5±5.3%) or milrinone (3.2±4.4%). The PIR was accompanied by a lusitropic response, and both were reversed by muscarinic receptor stimulation with carbachol and absent in the presence of β-AR blockade with timolol. OR-1896 inhibited PDE activity and increased cAMP levels at concentrations giving PIRs. OR-1896 did not sensitize the concentration-response relationship to extracellular Ca^2+^. Levosimendan, OR-1896 and EMD all increased the sensitivity to β-AR stimulation. The combination of either EMD and levosimendan or EMD and OR-1896 further sensitized the response, indicating at least two different mechanisms responsible for the sensitization. Only EMD sensitized the α_1_-AR response.

**Conclusion:**

The observed PIR to OR-1896 in rat ventricular strips is mediated through PDE3 inhibition, enhancing cAMP-mediated effects. These results further reinforce our previous finding that Ca^2+^ sensitization does not play a significant role in the inotropic (and lusitropic) effect of levosimendan, nor of its main metabolite OR-1896.

## Introduction

Levosimendan was synthesized to act as a positive inotropic agent for treatment of heart failure with increased Ca^2+^ sensitivity as the expected mechanism of action [1,2]. In addition, inhibition of phosphodiesterase (PDE) 3 has been described as a mechanism that could possibly contribute to the inotropic response. The relative contribution from each mechanism with respect to increased contractility has been a subject of substantial controversy. Surprisingly many authors have favoured a Ca^2+^ sensitising mechanism despite lacking consistency in the details necessary to explain a molecular interaction which could result in an inotropic response [3–10].

Potentially, Ca^2+^ sensitization of the myofilaments could increase contractility without raising intracellular Ca^2+^ levels, thus avoiding many of the detrimental effects of classical inotropic drugs, such as β-receptor agonists, e.g. dobutamine and PDE inhibitors, e.g. milrinone [11]. Although levosimendan is regarded to work through Ca^2+^ sensitization of the myofilaments with a minor additional contribution from inhibition of PDE3 [1,3,4,12], we recently published data demonstrating that the inotropic effect of levosimendan could be fully accounted for by PDE3 inhibition with no sign of contribution from Ca^2+^ sensitization. Some studies, however, have demonstrated long lasting hemodynamic effects of levosimendan administration several days after stop of infusion [13,14]. This prolonged effect is likely due to the active metabolite OR-1896, which has a half-life of up to 80 h [15]. An important question is whether this effect is mediated through enhanced Ca^2+^ sensitivity of the myofilaments, or if this effect can also be explained through inhibition of PDE3. Data from several studies suggest that the inotropic effects of OR-1896 are due to both increased Ca^2+^ sensitivity and PDE3 inhibition [10,16–18]. Currently, however, no studies have distinguished unequivocally between whether the positive inotropic response (PIR) to OR-1896 results from enhancing myofilament Ca^2+^ sensitivity or inhibition of PDE3.

Thus, the aim of the present study was to clarify the relative contributions of the Ca^2+^ sensitization and PDE3 inhibition to the functional effects of OR-1896 in rat myocardium, taking advantage of several experimental approaches. In this study we report that OR-1896 elicits an inotropic response that is mediated primarily, if not exclusively, through inhibition of PDE3. No Ca^2+^ sensitising component contributing to increased contractility could be found and the functional effects and mechanisms of action were very similar for OR-1896 and levosimendan.

## Materials and Methods

### Animals

Animal care was according to the Norwegian Animal Welfare Act, which conforms to the European Convention for the protection of Vertebrate animals used for Experimental and other Scientific Purposes (Council of Europe no. 123, Strasbourg 1985) and experiments were approved by the Norwegian Animal Research Authority. All studies are in accordance with the ARRIVE guidelines for reporting animal experiments [19,20].

### Preparation of rat ventricular muscle strips

Male Wistar rats of approximately 250–350 g were anaesthetized (2–3% isoflurane in air) and subsequently euthanized by cervical dislocation. The hearts were harvested and mounted on a Langendorff rig perfused with a relaxing solution containing (mM): NaCl (118.3), KCl (3.0), CaCl_2_ (0.2), MgSO_4_ (4.0), KH_2_PO_4_ (2.4), NaHCO_3_ (24.9), glucose (10.0) and mannitol (2.2). The left ventricle was exposed and ventricular strips were excised and mounted in organ baths.

### Measurement of ventricular strip contractility

Left ventricular strips from rat (approx. 1 mm diameter) were mounted in organ baths containing an oxygenated solution (31°C) as described above. After mounting the relaxing solution was replaced with a solution of identical composition with the exception of CaCl_2_ (1.8 mM) and MgSO_4_ (1.2 mM) concentrations. The muscles were field stimulated at a frequency of 1Hz with impulses of 5-ms duration and current about 20% above individual threshold (10–15 mA, determined in each experiment). The isometrically contracting muscles were stretched to the maximum of their length-tension curve [21]. Maximal developed force (F_max_), maximal development of force (dF/dt)_max_, time to peak force (TPF), time to 80% relaxation (TR80) and relaxation time (RT, defined as RT = TR80-TPF) were measured. The measurements were based on averaging 20–30 contraction-relaxation cycles (CRC). Inotropic responses were expressed as increases in (dF/dt)_max_ [21]. The descriptive parameters at the end of the equilibration period were used as basal (control) values. Blockers (added 90 min prior to agonist stimulation) of adrenergic (prazosin 100 nM, timolol 1 μM) and muscarinic cholinergic (atropine 1 μM) receptors were used unless otherwise indicated. Agonists were added cumulatively until the maximal response was obtained (concentration-response curves) or as a single bolus.

### Measurement of changes in the sensitivity of ventricular strips to extracellular Ca^2+^

In electrically stimulated strips, the contractile force is strictly dependent upon the concentration of extracellular Ca^2+^ determining the magnitude of the intracellular Ca^2+^ transient elicited by the electrical stimulation. After equilibration of the rat heart ventricular strips in 1.8 mM Ca^2+^ as described above, the Ca^2+^ concentration was reduced to 0.5 mM. When a new steady state was reached, the Ca^2+^ concentration was increased stepwise in a cumulative way to a maximum of 6 mM [22]. This procedure was performed in the absence or presence of OR-1896 or the Ca^2+^ sensitizer EMD57033. Concentration-response curves were constructed with maximum development of force set to 100%.

### Isolation of cardiomyocytes

Normal Wistar rat hearts were perfused (Langendorff set-up) with a Ca^2+^-free Joklik-MEM (Sigma-Aldrich, St. Louis, MO, USA) buffer and digested enzymatically by using collagenase type II (90 U·mL^−1^ final) (Worthington Biochemical Corp., Freehold, NJ, USA; 268 U·mg^−1^), as described previously [23].

### Fluorescence resonance energy transfer (FRET) imaging

Cardiomyocytes were attached to acid-washed and laminin-coated 24 mm glass coverslides in an 8-well dish and infected with the cAMP sensor RII_epac (generously provided in pAdEasy and previously characterized by Manuela Zaccolo [24,25]). Viruses were packaged, amplified and titer measured by VectorBiolabs (Philadelphia). Cardiomyocytes were infected and incubated with virus for 48h, using a MOI of 500. One coverslide was placed in a watertight imaging chamber (Attofluor; Life technologies) at room temperature with buffer A (mM): MgCl_2_ (1), KCl (1.97), KH_2_PO_4_ (0.43), K_2_HPO_4_ (1.5), CaCl_2_ (1), NaCl (144) and glucose (10). One to eleven cells were imaged in parallel and were visualized consecutively every 4 s at room temperature through a motorized digital inverted fluorescent microscope (iMIC; FEI Munich GmbH, Munich, Germany) with an air objective (10x NA0.4). Cells were excited at 436±10 nm and 500±10 nm for 20 ms consecutively using a monochromator (Polychrome V: FEI Munich), and emission from CFP and YFP were separated using a Dichrotome iMIC Dual Emission Module where a 515 nm LP filter separated the images from CFP and YFP onto a single EM-CCD camera chip (EVOLVE 512, Photometrix). Images were acquired by Live Acquisition browser (FEI Munich) and FRET was calculated using Offline Analysis (FEI Munich). FRET ratios were measured as ratios of YFP and CFP emission (F_530_/F_470_). YFP emission was corrected for direct excitations at 436 nm and spillover of CFP emission into the 530nm channel. The direct excitation of CFP at 500 nm and YFP emission at 470 nm was negligible.

### Phosphodiesterase assay

A modified standard two-step phosphodiesterase assay was performed [26]. Frozen tissue from rat left ventricle was homogenized prior to the addition of known concentrations of radiolabelled and unlabelled cAMP to a final concentration of 1 μM. There was a linear breakdown of cAMP for at least 20 min at 31°C. The reaction was stopped by heating for three minutes at 100°C. The second step of the assay was incubation with Snake Venom to convert 5’AMP to adenosine, which was not bound by Dowex added for separation. [^3^H]adenosine was counted in the supernatant. Dowex binds any unconverted cAMP and possible unconverted 5’AMP. Correctional steps take into account the binding ability of Dowex and the conversion efficiency (of [^14^C]5’-AMP) of Snake Venom.

### Drugs and reagents

Levosimendan was purchased from Zhou Xi Fen Pharm Chemical, Shanghai, China. The identity and purity of this levosimendan was validated by HPLC, MS and proton NMR spectroscopy, as previously described [27]. OR-1896 was purchased from Toronto Research Chemicals Inc., Toronto, Canada. Dowex anion exchange resin, adenosine 3′,5′-cyclic monophosphate, crotalus attrox venom, phenylmethanesulfonyl fluoride (PMSF), DL-Dithiothreitol, timolol maleate, isoprenaline hydrochloride, phenylephrine hydrochloride, carbachol (carbamoylcholine chloride), lidocaine hydrochloride and atropine were from Sigma-Aldrich (St. Louis, MO, USA). [^3^H]cAMP and [^14^C]5’-AMP were from PerkinElmer (Waltham, MA, USA). Cilostamide, rolipram and milrinone were from Tocris (Bristol, UK). EMD57033 was generously provided by Dr. Norbert Beier of Merck KGaA (Darmstadt, Germany).

### Statistics

Data are expressed as mean±SEM from n animals unless otherwise specified. P<0.05 was considered statistically significant (Student's t-test and ANOVA). When appropriate, Bonferroni corrections were made to control for multiple comparisons.

## Results

### OR-1896 evoked a positive inotropic response which was absent in the presence of PDE3 inhibition

**Fig 1:**
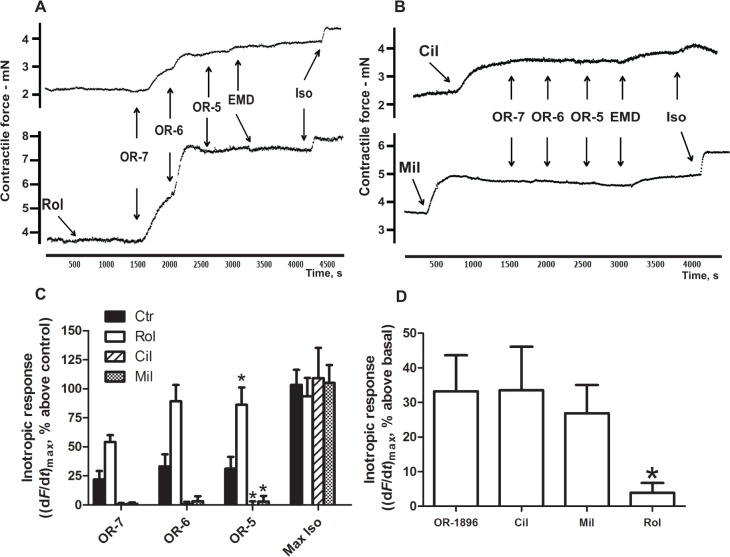
The inotropic response to OR-1896 is enhanced by PDE4 inhibition and absent in the presence of PDE3 inhibition. The figure shows representative original tracings of experiments showing the effect of increasing concentrations of OR-1896 (OR) alone and in the presence of PDE4 **(A)** and PDE3 **(B)** inhibitors. **A**) Effect of OR-1896 in increasing concentrations (OR-7: 100 nM, OR-6: 1 μM and OR-5: 10 μM, n = 6) and the effect of OR-1896 in the presence of the PDE4 inhibitor rolipram (Rol, 10 μM, n = 6). **B)** Effect of OR-1896 in the presence of a PDE3 inhibitor, either cilostamide (Cil, 1 μM, n = 6) or milrinone (Mil, 1 μM). **A, B)** EMD57033 (EMD, 3 μM) was added after OR-5. Isoprenaline (Iso, 100 μM) administered at the end gave the maximum inotropic response achievable in the strip. **C)** Bar graph showing the inotropic response to different concentrations of OR-1896 in the presence and absence (Ctr) of PDE inhibitors in rat myocardial strips (n = 6). **D)** Bar graph comparing the inotropic response of OR-1896 (1 μM) to the inotropic response of different PDE inhibitors in rat ventricular strips (n = 6). All experiments were conducted on rat ventricular muscle strips in the absence of timolol and in the presence of α_1_-AR (prazosin, 100 nM) and muscarinic receptor blockade (atropine, 1 μM). Basal force values for each group (mN): OR-1896: 4.3±0.7; Rol: 3.5±0.5; Cil: 3.2±0.5; Mil: 2.6±0.3.

We measured the contractile response to OR-1896 in normal rat heart ventricular strips. In the absence of β-AR blockade, OR-1896 evoked a concentration-dependent PIR, reaching a maximum of 33±10% above basal at 1 μM (Fig. 1A, 1C and 1D). The PDE4 inhibitor rolipram (Rol, 10 μM, n = 6) enhanced the PIR to OR-1896, peaking at 89±14% above control (Fig. 1A and 1C, n = 6). The PIR to OR-1896 was barely detectable in the presence of PDE3 inhibition by cilostamide (Cil, 1 μM) or milrinone (Mil, 1 μM) (Fig. 1B and 1C, n = 6). The Ca^2+^ sensitising agent EMD57033 (EMD, 3 μM) evoked a PIR on top of OR-1896 alone of 9±3% of basal, and similarly evoked a PIR on top of OR-1896 in the presence of Cil (EMD response: 11±3% of basal) or Mil (EMD response: 12±2% of basal). In the presence of Rol and OR-1896, EMD evoked a small PIR of 3±3% of basal; the effect was smaller most likely due to some strips having obtained the maximum PIR (Fig. 1A and 1B). Isoprenaline (10^–4^ M) given at the end of the experiment demonstrated that all groups had additional capacity to elicit a PIR.

### OR-1896 evoked a positive inotropic response similar to that evoked by PDE3 inhibition

In the absence of β-AR blockade in ventricular strips, OR-1896 (1 μM, n = 6) evoked a PIR similar to that evoked by the PDE3 inhibitors cilostamide (Cil, 1 μM, n = 6) and milrinone (Mil, 1 μM, n = 6) (OR-1896: 33±10% above basal, Cil: 34±13% above basal, Mil: 27±8% above basal, Fig. 1D). Inhibition of PDE4 by rolipram (Rol 10 μM, n = 6) evoked a small PIR compared to OR-1896 (Rol: 3.9±3.0% above basal, p<0.05 vs. OR-1896, Fig. 1D).

### OR-1896 and PDE3 inhibitors elicited similar lusitropic responses

**Fig 2:**
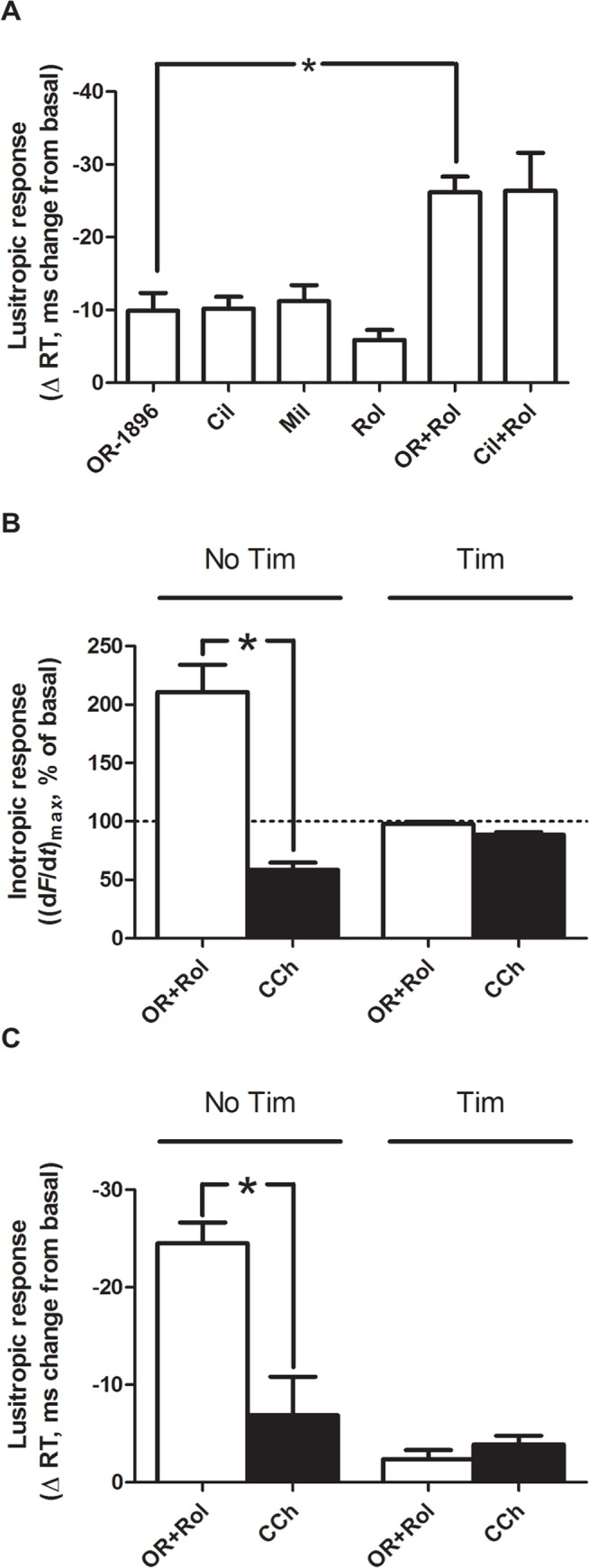
The OR-1896-evoked inotropic response is associated with a lusitropic response with similar characteristics of cAMP-dependence. **A)** Lusitropic response, shown as the change in relaxation time (RT) compared to basal elicited by OR-1896 and known PDE inhibitors in rat ventricular strips in the absence of timolol (n = 6). **B&C)** Bar graphs showing the effect of carbachol (CCh, 20 μM) on the inotropic and lusitropic response to combined OR-1896 (1 μM) and rolipram stimulation in rat ventricular strips (n = 6 strips from 3 rats). The results were compared to a group which received timolol (Tim) prior to OR-1896 and rolipram (n = 6 strips from 3 rats). Basal force values (mN): Ctr: 5.0±1.1 vs Tim: 3.7±1.2 **A, B, C)** PDE4 inhibitor: Rolipram (Rol, 10 μM), **A)** PDE3 inhibitors: cilostamide (Cil, 1 μM), milrinone (Mil, 1 μM). All data are mean ± SEM, * = p<0.05.

We previously reported that the PIR of levosimendan was accompanied by a lusitropic effect (reduction of RT) indicative of cAMP-dependency [27]. In rat ventricular strips, this effect was relatively small when levosimendan was administered alone, but significantly larger in the presence of PDE4 inhibition. Similarly, in the absence of β-AR blockade, OR-1896 in rat left ventricular strips demonstrated a modest reduction in RT (-9.9±2.7 ms vs. basal, n = 6), an effect very similar in magnitude to that of Cil (-10.1±1.8 ms vs. basal, n = 6) and Mil (-11.2±2.2 ms vs. basal, n = 6, Fig. 2A). The lusitropic effect of OR-1896 (1–10 μM) was also attenuated in the presence of both Cil and Mil (data not shown). In the presence of PDE4 inhibition by Rol, the lusitropic effect of OR-1896 was significantly enhanced compared to OR-1896 alone (-26.1±2.3 ms vs. basal, n = 6, p<0.05). This was comparable with the lusitropic response to combined Cil and Rol (-26.3±5.2 ms vs. basal, n = 6, Fig. 2A).

### The OR-1896-evoked inotropic and lusitropic response was reversed by muscarinic receptor stimulation with carbachol

In the absence of atropine and β-AR blockade in rat ventricular strips, combined OR-1896 (OR, 1 μM) and rolipram (Rol, 10 μM) evoked a large PIR of 211±33% of basal value (n = 6 strips from 3 animals; p<0.05). This response was completely reversed by the muscarinic receptor agonist, carbachol (CCh, 20 μM; p<0.05; Fig. 2B). Similarly, combined OR-1896 and Rol evoked a large lusitropic effect of 24.5±3 ms, an effect also reversed by carbachol (CCh, 20 μM; n = 6 strips from 3 animals; p<0.05; Fig. 2C). In the presence of β-AR blockade (timolol, 1 μM), combined OR-1896 and Rol did not evoke an inotropic or lusitropic response (n = 6 strips from 3 animals; Fig. 2B and 2C). No significant alterations to basal force between the two groups could be observed (basal force values (mN): Ctr: 5.0±1.1 vs timolol: 3.7±1.2). Thus, both the inotropic and lusitropic response to OR-1896 appears dependent upon simultaneous β-AR stimulation (here by endogenous noradrenaline, released by electrical stimulation).

### OR-1896 did not alter the concentration-response relationship of Ca^2+^

**Fig 3:**
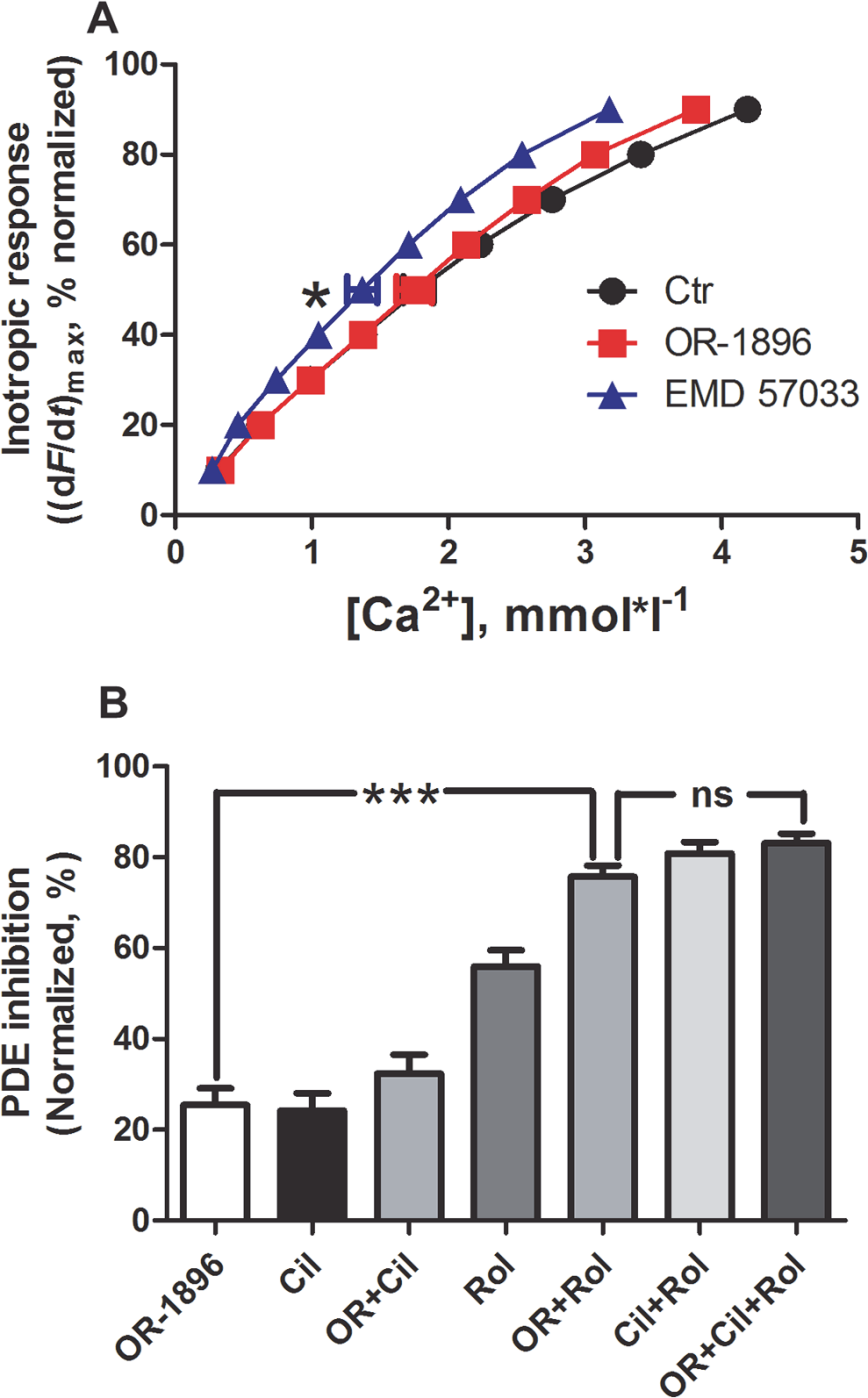
A) Lack of Ca^2+^ sensitization by OR-1896. The effect of EMD57033 (EMD, 3 μM, n = 7) or OR-1896 (1 μM, n = 7)) on the Ca^2+^ concentration-response relationship. Basal force values (mN): Ctr: 2.5±0.3 vs. OR-1896: 2.4±0.3. Mean ± SEM, * p<0.05. B) PDE3 inhibition by OR-1896. Effect of OR-1896 (1 μM) and PDE inhibitors (Cilostamide, Cil 1 μM; Rolipram, Rol 10 μM) upon PDE activity in homogenates of normal rat heart ventricle. Mean ± SEM, *** = p<0.005, ns = non-significant.

If OR-1896 was a Ca^2+^ sensitizer, it would be expected to influence the concentration-response relationship of Ca^2+^ in dynamically contracting ventricular strips. We therefore conducted experiments on the concentration-response relationship to Ca^2+^ in electrically stimulated ventricular strips in the presence and absence of OR-1896. Under these conditions OR-1896 did not sensitize the concentration-response relationship to Ca^2+^ (EC_50_: OR-1896: 1.8±0.1 mM Ca^2+^ (n = 7) vs. Ctr: 1.8±0.1 mM Ca^2+^ (n = 7); Fig. 3A). In contrast, the established Ca^2+^ sensitizer EMD caused a significant increase in the Ca^2+^ sensitivity in this preparation (EC_50_: 1.4±0.1 mM Ca^2+^ (n = 7), vs. Ctr: 1.8±0.1 mM Ca^2+^, p<0.05; Fig. 3A).

### OR-1896 inhibited PDE3 to a similar extent as cilostamide

To verify that OR-1896 was able to inhibit PDEs, we conducted PDE activity assays on homogenized tissue from normal rat heart (Fig. 3B). OR-1896 at 1 μM, the concentration that induced the maximum PIR, inhibited PDE activity by 26±4%, n = 6, comparable to the PDE3 inhibitor, cilostamide, Cil (24±4%, n = 5). The PDE4 inhibitor rolipram (Rol) inhibited PDE activity by 56±4%, and OR-1896+Rol in combination inhibited the activity by 76±3%. Inhibition of both PDE3 and PDE4 together (with Rol and Cil) inhibited 81±2% of total PDE activity (Cil+Rol, n = 6). The addition of OR-1896 in addition to PDE3 and PDE4 inhibition did not yield further inhibition of PDE activity (Cil+Rol+OR: 83±2.0%, n = 6). Thus, the effects of OR-1896 and Cil were similar and mutually exclusive.

### OR-1896 increased cAMP levels in rat isolated left ventricular cardiomyocytes

**Fig 4:**
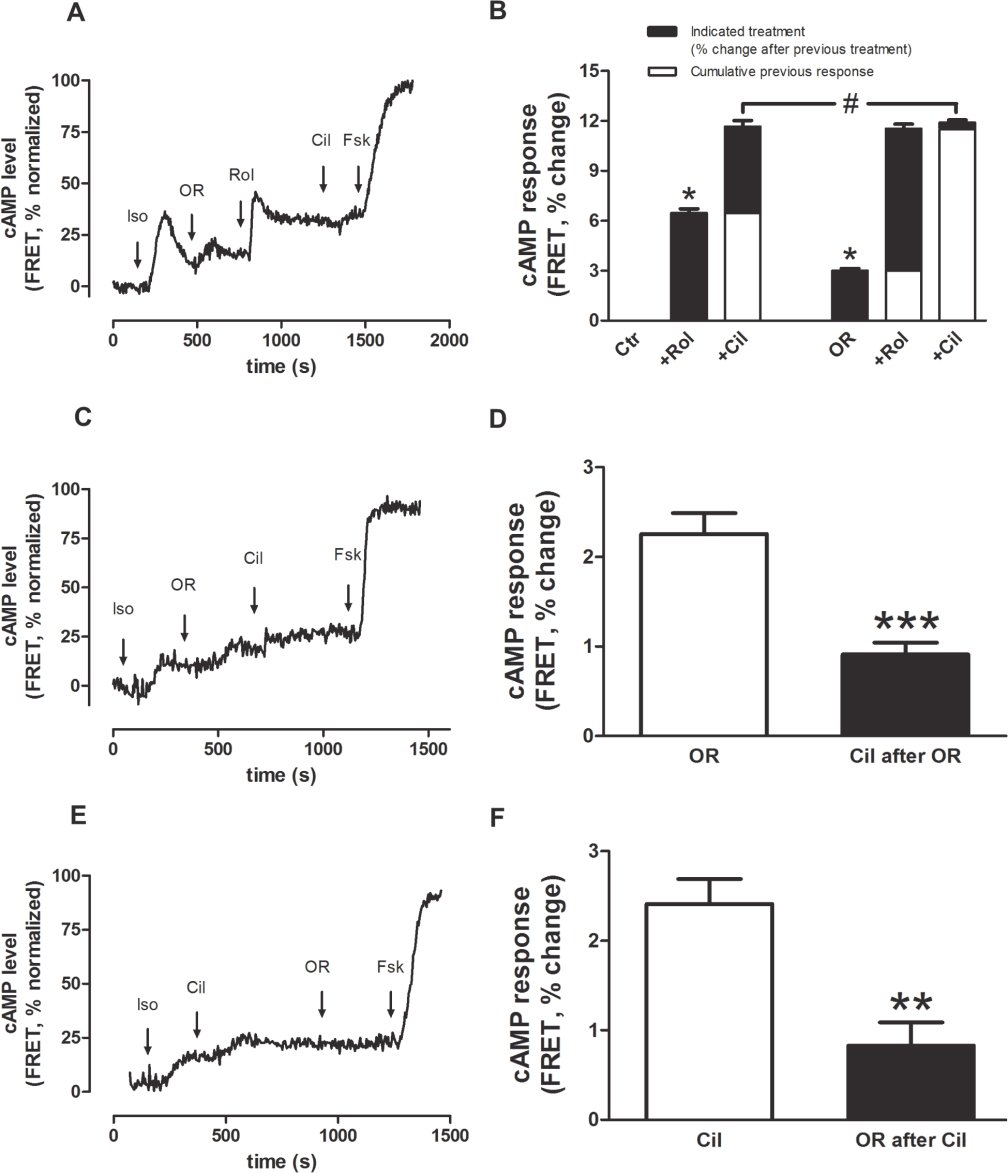
OR-1896 causes cAMP increase by PDE3 inhibition. A, C, E) Experimental readouts of cAMP measured as the FRET signal from the RII_epac sensor in isolated cardiomyocytes. All cells were primed with a small concentration of isoprenaline (5 nM) prior to PDE inhibition. Forskolin (Fsk, 3 μM) was administered at the end as a measure of the maximum achievable FRET response, indicating that the sensors were not fully saturated during measurements. The readouts are normalized to the maximal response to Fsk. **B)** Bar graph showing the FRET response to OR-1896 (OR, 1 μM), followed by rolipram (Rol, 10 μM) and the attenuated response to cilostamide (Cil, 1 μM) following OR-1896 + Rol compared to Rol alone (n = 38 cells from 3 animals) The FRET responses are compared to a set of cells that did not receive OR-1896 (Ctr). **D)** Bar graph showing the FRET response to OR-1896 and the attenuated response to Cil after OR-1896 (n = 26 cells from 2 animals). **F)** Bar graph showing the FRET response to Cil and the attenuated response to OR-1896 after Cil (n = 24 cells from 2 animals). Data are mean ± SEM, * = p<0.05, ** = p<0.05 compared to OR-1896 (Fig. 4D), *** = p<0.05 compared to Cil (Fig. 4F), # = p<0.005.

OR-1896 increased cAMP levels in rat isolated left ventricular cardiomyocytes as detected by the RII_epac cAMP sensor (Fig. 4). After an initial stimulation with 5 nM isoprenaline (Iso) (FRET response Iso: 8.1±0.5% above basal), OR-1896 (1 μM) induced a further FRET response of 3.0±0.2% (Fig. 4A and 4B) and enhanced the subsequent effect of PDE4 inhibition by rolipram (Rol) (FRET response Rol: 8.5±0.5% after OR-1896 vs. 6.5±0.4% in its absence, p<0.05; Fig. 4A and 4B). Summation of the FRET response to OR-1896 and Rol was comparable to that of cilostamide (Cil) and Rol (FRET response OR-1896 +Rol: 11.5±0.5% vs Cil+Rol: 11.7±0.8%). The FRET response to Cil was significantly reduced in the presence of OR-1896+Rol (FRET response: 0.4±0.3%) compared to the response to Cil in the presence of Rol, but absence of OR-1896 (FRET response: 6.5±0.4), p<0.05; Fig. 4B. These results would be expected if the effect of OR-1896 is caused by PDE3 inhibition in a manner similar to Cil. As illustrated in Fig. 4C and E and quantified in Fig. 4D and F, OR-1896 and Cil had comparable effects (2.3±0.2% vs. 2.4±0.2% after Iso) and mutually attenuated the response to each other (Cil after OR-1896: 0.9±0.1%; OR-1896 after Cil: 0.8±0.2%, both p<0.05 vs. the effect of Cil or OR-1896), consistent with their effects being mediated by a similar mechanism.

### OR-1896 and levosimendan both sensitized responsiveness through a receptor system which increases cAMP, but neither sensitized a cAMP independent receptor system

If OR-1896 exerts effects at least in part through Ca^2+^ sensitization, it may potentiate effects through receptor systems enhancing Ca^2+^ sensitivity. Similarly, if OR-1896 operates through a PDE3 inhibitory mechanism, cAMP-dependent receptor signalling should be potentiated or enhanced [28]. Therefore, we studied the effect of OR-1896 on both a cAMP-activating receptor system (β-AR activation) and a cAMP independent receptor system (α_1_-AR activation) and compared it with levosimendan, which we had studied previously [27].

**Fig 5:**
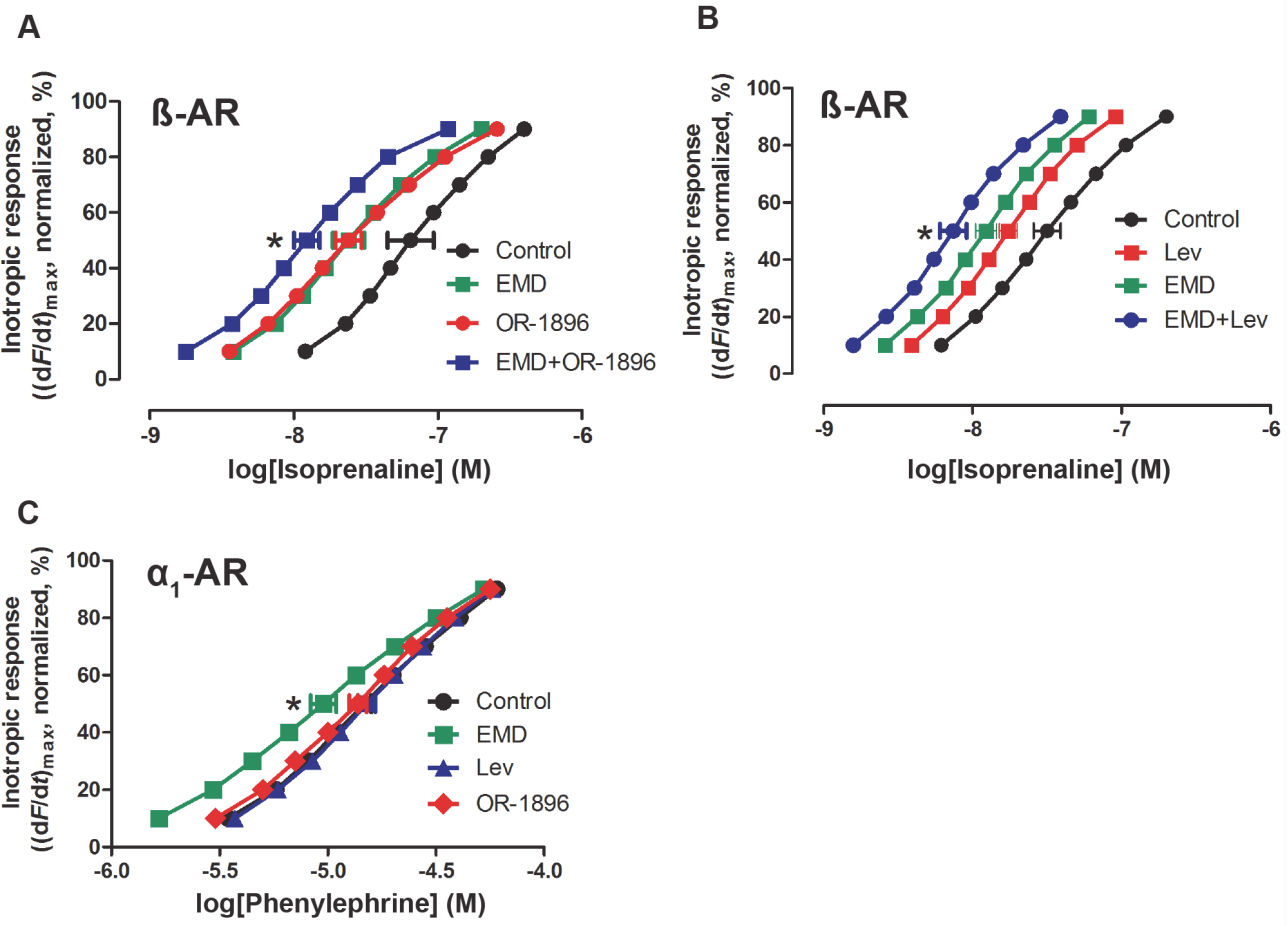
Effect of OR-1896, levosimendan and the Ca^2+^ sensitizer EMD57033 on concentration-response curves to β- and α_1_-adrenoceptor stimulation of inotropic response in rat ventricular strips. **A)** The effect of OR-1896 (1 μM, n = 7) and EMD57033 (EMD, 3 μM, n = 7) alone and in combination (n = 7) on the concentration-response curves of isoprenaline in the presence of α_1_-adrenergic (prazosin, 100 nM) and muscarinic (atropine, 1 μM) receptor blockade. Basal force values (mN): Ctr: 3.9±0.3, OR-1896: 3.1±0.4, EMD: 3.4±0.4, EMD+OR-1896: 3.4±0.3. **B)** The effect of levosimendan (Lev, 1 μM, n = 14) and EMD alone (n = 12) and in combination (n = 7) on the concentration-response curves of isoprenaline in the presence of α_1_-adrenergic and muscarinic receptor blockade. Basal force values (mN): Ctr: 3.9±0.4, Lev: 3.7±0.4, EMD: 3.7±0.3, EMD+Lev: 3.2±0.4. **C)** The effect of levosimendan (1 μM, n = 7), OR-1896 (1 μM, n = 15) and EMD (3 μM, n = 14) on the concentration-response curves of phenylephrine in the presence of β-adrenergic (timolol, 1 μM) and muscarinic receptor blockade. Basal force values (mN): Ctr: 4.2±0.5, Lev: 4.0±0.3, OR-1896: 4.5±0.3, EMD: 3.5±0.4. Data are mean ± SEM, * = p<0.05.

In normal rat ventricular strips, in the presence of α_1_-AR inhibition, the concentration-response relationship to isoprenaline (β-AR system) was potentiated by OR-1896 (pEC_50_: OR-1896: 7.6±0.1 (n = 7) vs. Ctr: 7.2±0.2 (n = 7), p<0.05; Fig. 5A). In the same set, the Ca^2+^ sensitizer EMD also sensitized the response to isoprenaline (pEC_50_: EMD: 7.6±0.1 (n = 7), p<0.05 vs. Ctr; Fig. 5A). The combination of EMD and OR-1896 further sensitized the response to isoprenaline beyond that of EMD (pEC_50_: EMD+OR-1896: 7.9±0.1 (n = 7), p<0.05 vs. EMD; Fig. 5A).

In another set of experiments levosimendan sensitized the response to isoprenaline (pEC_50_: Lev: 7.8±0.1 (n = 14) vs. Ctr: 7.5±0.1 (n = 14), p<0.05; Fig. 5B). EMD also sensitized the concentration-response relationship to isoprenaline (pEC_50_: EMD: 7.9±0.1 (n = 12), p<0.05 vs. Ctr). The combination of EMD and levosimendan further sensitized the concentration-response relationship to isoprenaline beyond that of EMD alone (pEC_50_: EMD+Lev: 8.1±0.1, n = 7, p<0.05 vs. EMD, Fig. 5B).

Stimulation of rat left ventricular strips with phenylephrine (α_1_-AR agonist, timolol present) in the presence and absence of EMD, levosimendan and OR-1896, revealed that only EMD sensitized the response (pEC_50_ EMD: 5.0±0.1 (n = 14) vs. Ctr: 4.8±0.1 (n = 21), p<0.05) (Fig. 5C). Neither levosimendan nor OR-1896 altered the EC_50_ to phenylephrine (pEC_50_ Lev: 4.8±0.1 (n = 6) vs. OR-1896: 4.9±0.1 (n = 15), both ns vs. Ctr; Fig. 5C).

## Discussion

In this paper, we provide strong support that in rat left ventricle the PIR to OR-1896, the active metabolite of levosimendan, results primarily, if not exclusively, from inhibition of PDE3. This conclusion is drawn from the primary findings that 1) OR-1896 did not evoke a PIR in the presence of PDE3 inhibition and 2) OR-1896 did not increase functional Ca^2+^ sensitivity in the dynamic contractile model used in these studies. In addition, several characteristics of the OR-1896 PIR indicate it is cAMP-mediated. In particular, the PIR is accompanied by a lusitropic response and both responses are sensitive to a reduction of cAMP levels mediated by either muscarinic receptor stimulation (carbachol) or β-AR blockade (timolol). The findings in this study correspond well in all aspects to our prior study on levosimendan in human and rat myocardium [27], further strengthening our hypothesis that levosimendan-evoked PIR results from inhibition of PDE3. These findings are highly relevant, since some studies proclaim the clinical effects of levosimendan at least in part result days later from increased serum levels of OR-1896 increasing Ca^2+^ sensitivity [13,14].

The data in this study are consistent with other reports that both levosimendan [3–5,12,29] and OR-1896 [16,17] evoke a PIR, increase the level of cAMP [3,5] and inhibit PDE3 [10,29]. Whereas these reports also observed effects upon Ca^2+^ sensitization [12,16,30], we found no functional evidence that levosimendan or OR-1896 evoked PIRs through Ca^2+^ sensitization in our model. It is important to clarify with certainty if levosimendan and OR-1896 evoke PIRs through cAMP elevation, because evoking PIRs in this manner is considered detrimental and linked to increased morbidity and mortality in heart failure [11].

### OR-1896 did not evoke an inotropic response when PDE3 was inhibited

In ventricular strips where either of two separate PDE3 inhibitors (cilostamide or milrinone) was present, OR-1896 did not evoke a PIR. On the other hand, PDE4 inhibition (rolipram) enhanced the effect of OR-1896. This enhanced PIR is consistent with a previously reported synergistic effect of PDE3 and PDE4 inhibition [31], and could indicate that OR-1896 had powerful inhibitory effects upon PDE3. Directly assessing PDE activity in rat ventricular heart homogenate, we confirmed that OR-1896 at a concentration which gave an inotropic response significantly inhibited PDE3 activity. These findings are consistent with the results of Szilagyi *et al*. [10] that reported 1 μM OR-1896 to inhibit ∼80% of PDE3 activity, whereas no inhibitory effect was observed upon PDE4 in guinea pig heart. In fact, Szilagyi *et al*. show a good correlation between the concentration range in which levosimendan and OR-1896 inhibit PDE3 and the relative force increase in permeabilized guinea pig myocytes. It would have been interesting to know if prior cilostamide treatment would have attenuated the levosimendan and OR-1896 inotropic responses in their models as it did in ours.

### OR-1896 evoked no visible increase in Ca^2+^ sensitivity in the presence of increased concentrations of extracellular Ca^2+^

Studies have established that levosimendan [8,10,27] and OR-1896 [10] undoubtedly inhibit PDE3 at concentrations known to elicit PIRs. Therefore, it is an absolute requisite to conduct studies that measure levosimendan or OR-1896-mediated increases in Ca^2+^ sensitivity simultaneously with a PIR in the presence of prior PDE3 inhibition. It is important that such a study is conducted in a model with dynamic handling of Ca^2+^, such as the one in the present paper. Studies showing Ca^2+^ sensitization in a steady state model without dynamic Ca^2+^ handling (e.g. skinned fibres) will not necessarily represent a true physiological effect of levosimendan or OR-1896, thus weakening the translational value to contractility. In this study we found no apparent Ca^2+^ sensitization of the myofilaments by OR-1896 when concentration-response relationships to Ca^2+^ were conducted. These findings do not detract from the fact that levosimendan and OR-1896 could sensitize the myofilaments to Ca^2+^ under certain conditions (e.g. skinned fibres and molecular studies). Furthermore, because we do not measure intracellular levels of Ca^2+^, we cannot exclude a potential Ca^2+^ sensitizing mechanism occurring within the cardiomyocyte in our model. However, our results give strong indication that in our model in rat ventricular myocardium, Ca^2+^ sensitization (if present) does not appear to have any translational value to the PIR. If Ca^2+^ sensitization of the myofilaments by OR-1896 is sufficient to mediate an inotropic response alone, this inotropic effect should still be present in the presence of PDE3 inhibition. To confirm that our model could measure effects presumed to result from increased Ca^2+^ sensitivity, we studied EMD 57033. Although OR-1896 and EMD 57033 both bind to troponin C, the latter has been proposed to also bind to a site on myosin that facilitates the actin-myosin interaction possibly altering the ATPase activity [32]. This means the mechanism for potentially evoking Ca^2+^ sensitivity of the myofilaments by EMD and OR-1896 is comparable but not identical. Under prior PDE3 inhibition, EMD further increased the PIR and it also sensitized the response to both α_1_-AR stimulation and the response to cumulative concentrations of Ca^2+^, neither of which were influenced by OR-1896. This demonstrates at least in part that a potential Ca^2+^ sensitizer can evoke PIRs which are independent of cAMP accumulation. Thus, even though OR-1896 may increase Ca^2+^ sensitivity in our model, it does not appear to contribute to the PIR.

### OR-1896 increased levels of cAMP in the concentration range corresponding with the inotropic response

It has been reported that combined PDE3 and 4 inhibition increased contractile force in rat ventricle through increasing levels of cAMP [31]. We observed a similar phenomenon when combining OR-1896 and rolipram. If PDE3 inhibition is responsible for the synergistic inotropic effect of combining OR-1896 and rolipram, one would expect increased levels of cAMP. Accordingly, levosimendan has been reported to increase levels of cAMP in guinea pig heart tissue [5] and guinea pig cardiomyocytes [3]. Using the RII_epac FRET sensor, previously shown to measure cAMP close to phospholamban and troponin I [25], we found that OR-1896 also significantly increased cAMP levels. Cilostamide given prior to OR-1896 attenuated this cAMP increase, implying that the cAMP increase resulted from PDE3 inhibition. Although the OR-1896-evoked increase in cAMP levels measured by FRET was modest, it was approximately the same as that evoked by cilostamide.

### The OR-1896-evoked inotropic and lusitropic effects were attenuated by muscarinic receptor stimulation with carbachol and were dependent on β-AR activation

In our model OR-1896 evoked very similar lusitropic effects as cilostamide and milrinone. This is an important finding and corresponds with other studies in different animal models [16,17]. The association of an inotropic response with a lusitropic response, revealed as earlier onset of relaxation (reduction of time to peak force (TPF)) and increased rate of relaxation (reduction of RT), giving a shortening of the contraction-relaxation cycle, is a hallmark of activation of the cAMP/PKA pathway [21]. In our model all experiments revealed synchronous inotropic and lusitropic responses indicative of a common mechanism. This was visible when comparing both the inotropic and lusitropic effects of OR-1896 in the presence of different PDE inhibitors and was particularly obvious when studying the effect of carbachol on the inotropic and lusitropic responses to OR-1896. Previously, others have demonstrated that the inotropic response to both levosimendan and OR-1896 is markedly attenuated or absent in the presence of carbachol [3,16,33]. Here we report that similar to the inotropic response, the lusitropic response to OR-1896 was also sensitive to carbachol. Similar findings have been reported by others in canine myocardium [17], and this sensitivity to carbachol corresponds well with previous reports on cAMP-mediated responses [34]. Although the reversal of the lusitropic response was incomplete compared to the inotropic response, this is a typical finding (see e.g. [34]) and may be due to slow reversal kinetics of protein phosphorylation involved in the lusitropic response, for instance slow dephosphorylation of troponin I [35–37].

In our model, activation of the β-AR signalling pathway appears to play a crucial role in the response to OR-1896. Correspondingly, we report that OR-1896 sensitized β-AR stimulation, gave lusitropic effects similar to cilostamide and no inotropic or lusitropic effect in the presence of timolol. This corresponds with other studies; Boknik *et al*. [3] reported that levosimendan sensitized β-AR stimulation in guinea pig hearts, and Haikala *et al*. [7] observed an attenuated response to levosimendan in the presence of timolol. These effects of OR-1896 and levosimendan could be a result of a model relying heavily on β-AR activation to induce inotropic effects. It is well established that β-AR stimulation suppresses myofilament Ca^2+^ sensitivity through phosphorylation of troponin I, which may antagonize the increase in Ca^2+^ sensitivity induced by OR-1896. In this respect any Ca^2+^ sensitization by OR-1896 must be more labile than by EMD under the current experimental conditions. Alternatively, levosimendan- and OR-1896-mediated inotropic effects result primarily from increased levels of cAMP. In this case the latter appears to have more support in models directly measuring contractile force, since data are consistent across several different animal models [10,16,18].

### Limitations

Although our findings support PDE3 inhibition as the mechanism of action responsible for the levosimendan- and OR-1896-evoked PIR, there are some limitations to this study that warrant consideration: 1) we did not measure intracellular Ca^2+^ levels, and although our animal model does not reveal any functional effects beyond PDE3 inhibition, we cannot exclude that OR-1896 enhances Ca^2+^ sensitivity within the cardiomyocyte. Nonetheless, assuming OR-1896 enhances Ca^2+^ sensitivity, this mechanism appears to play a negligible role in the OR-1896-evoked PIR; 2) there is a variety of species-dependent variation in the PIR evoked by levosimendan and OR-1896; 3) as such, rat ventricular strips may be unique in that the PIR of OR-1896 appears to be primarily dependent upon enhancing the effect of endogenous noradrenaline. This differs from most other species, in which OR-1896 as well as PDE3 inhibitors elicit their PIRs even in the presence of β-blockers; and 4) the mechanism mediating the PIR of levosimendan and OR-1896 in vivo could be influenced by effects which are independent of both PDE3 inhibition and Ca^2+^ sensitization [7,38]. It is known that levosimendan opens ATP-dependent potassium channels (leading to vasodilation and decreased afterload), mitochondrial protection, anti-inflammatory and anti-apoptotic effects [39]. These effects are not measured and accounted for in this study and could indirectly contribute to an apparent increase in contractility and contribute to the clinical findings in both human and animal studies. It is not known if OR-1896 also evokes the same mechanisms and effects.

### Conclusions

Despite these limitations, the data indicate, at least in rat left ventricular myocardium, that the PIR to both levosimendan and OR-1896 is evoked primarily, if not exclusively, through increasing cAMP levels by inhibition of PDE3.
